# Assessment tools for determining appropriateness of admission to acute care of persons transferred from long-term care facilities: a systematic review

**DOI:** 10.1186/1471-2318-14-80

**Published:** 2014-06-22

**Authors:** Anna Renom-Guiteras, Lisbeth Uhrenfeldt, Gabriele Meyer, Eva Mann

**Affiliations:** 1 School of Nursing Science, Faculty of Health, University of Witten/Herdecke, Witten, Germany; 2 Universitat Autònoma de Barcelona, Barcelona, Spain; 3 Institute of General Practice and Family Medicine, Faculty of Health, University of Witten/Herdecke, Alfred-Herrhausen-Str. 50, Witten D-58448, Germany; 4 Department of Research, Horsens Hospital, Department of Public Health, Aarhus University, Aarhus, Denmark; 5 Medical Faculty, Institute of Health and Nursing Science, Martin-Luther-University Halle-Wittenberg, Magdeburger Straße 8, Halle (Saale) D-06112, Germany; 6 Institute of General Practice, Family Medicine and Preventive Medicine, Paracelsus Medical University, Salzburg, Austria

**Keywords:** Nursing home, Patient transfer, Hospitalization, Systematic review

## Abstract

**Background:**

Residents of long-term care facilities have a high risk of acute care admission. Estimates of the frequency of inappropriate transfers vary substantially throughout the studies and various assessment tools have been used. The purpose of this study is to systematically review and describe the internationally existing assessment tools used for determining appropriateness of hospital admissions among long-term care residents.

**Method:**

Systematic review of the literature of two databases (PubMed and CINAHL®). The search covered seven languages and the period between January 2000 and December 2012. All quantitative studies were included if any assessment tool for appropriateness of hospital and/or emergency department admission of long-term care residents was used. Two pairs of independent researchers extracted the data.

**Results:**

Twenty-nine articles were included, covering study periods between 1991 and 2009. The proportion of admissions considered as inappropriate ranged from 2% to 77%. Throughout the studies, 16 different assessment tools were used; all were based on expert opinion to some extent; six also took into account published literature or interpretation of patient data. Variation between tools depended on the concepts studied, format and application, and aspects evaluated. Overall, the assessment tools covered six aspects: specific medical diagnoses (assessed by n = 8 tools), acuteness/severity of symptoms (n = 7), residents’ characteristics prior to admission (n = 6), residents’ or families’ wishes (n = 3), existence of a care plan (n = 1), and availability or requirement of resources (n = 10). Most tools judged appropriateness based on one fulfilled item; five tools judged appropriateness based on a balance of aspects. Five tools covered only one of these aspects and only six considered four or more aspects. Little information was available on the psychometric properties of the tools.

**Conclusions:**

Most assessment tools are not comprehensive and do not take into account residents’ individual aspects, such as characteristics of residents prior to admission and wishes of residents or families. The generalizability of the existing tools is unknown. Further research is needed to develop a tool that is evidence-based, comprehensive and generalizable to different regions or countries in order to assess the appropriateness of hospital admissions among long-term care residents.

## Background

Residents of long-term care (LTC) facilities have a high risk of being admitted to hospital. Internationally, the incidence of visits to an emergency department has been estimated to be approximately 30 transfers per 100 LTC beds per year [1]. LTC residents are often sent to emergency departments (ED) when they are in a highly acute condition, and are likely to be admitted to the hospital [2]. Common underlying diagnoses are pneumonia, urinary tract infection, congestive heart failure, chronic obstructive pulmonary disease, fall-related injuries, and altered conscious state [3,4].

LTC residents are often frail and suffer from diseases in advanced stages, have several comorbidities, high levels of dependency and take multiple medications. The referral or admission to an ED or acute hospital – although often unavoidable and beneficial – represents an unfavourable discontinuity of care and encompasses threats to the residents including distress, risk of iatrogenic events [5], and deterioration of mobility and cognition [6,7]. Beyond adverse clinical effects, hospital transfers account for a high proportion of total healthcare costs [8].

Many authors have evaluated the appropriateness of ED visits or hospitalisation among LTC residents. There is an on-going debate on how to define appropriateness of admissions in order to reduce negative effects of inappropriate transfers without withholding residents from admission if acute care is needed. To distinguish between admissions to acute care that are inappropriate and those that are not is of great interest not only for the residents concerned but also for nursing home providers and policy makers alike. In international studies, between 10% and 60% of hospital admissions have been classified as inappropriate [9,10]. So far, the reason for this high variability is not clarified. Variations may result from different study objectives, including different concepts such as inappropriate, preventable, avoidable, or unnecessary hospitalisation. Differences in acute care destinations and nursing home populations included in the studies may also affect the rates of inappropriate admissions. Several studies suggest that facility characteristics may be as important as residents’ clinical characteristics [11,12]. In addition, regional differences in terms of financial incentives may also have an influence [13]. Interestingly, considerable variations in inappropriate hospital admission rates were even found in studies including nursing homes in well-defined areas only [14].

It is also important to take into account that authors used different assessment tools to judge the appropriateness of acute care transfers. Up to now, there is no consensus on which tool to use for assessment of appropriateness of residents’ hospital admission. Furthermore, there is no agreement on the aspects to be covered by such a tool. The terminology and definitions are not yet clarified, as claimed by some authors [11,15-17]. As a first step towards clarification, it seems to be justified to systematically review all assessment instruments applied for judgement of appropriateness of transfers, to analyse their development, their underlying concepts, the aspects included, their psychometric properties, and to critically review them in the context of the complexity of acute care admissions of frail and vulnerable LTC residents.

Thus, the aim of our systematic review is 1) to provide an overview of the studies dealing with tools for assessing appropriateness of hospital admissions in LTC residents and 2) to describe the published assessment tools in detail, including information about their development and the aspects covered by the tools.

## Methods

Four researchers from Spain, Germany, Denmark and Austria, all experienced in geriatric care and research, established a working group and developed a research protocol (available from the authors on request). In January 2013, two reviewers conducted a literature search. The search covered the databases Medline via PubMed and CINAHL® and was limited to studies published between January 2000 and December 2012. The following search strategy was used for Pubmed: (("Residential Facilities"[MeSH]) OR (nursing homes) OR (homes for the aged) OR (aged care facilit*) OR (nursing facilit*) OR ("Long-Term Care"[MeSH])) AND (("Emergency Service, Hospital"[MeSH]) OR hospital OR (acute care) OR (emergency AND (medicine OR department* OR unit* OR ward* OR service* OR room*))) AND (appropriat* OR suitable OR avoidable OR preventable) AND (("Patient Transfer"[MeSH]) OR ("Hospitalization"[MeSH]) OR referral* OR admission* OR transition*) AND (English[lang] OR French[lang] OR German[lang] OR Spanish[lang] OR Catalan[lang] OR Danish[lang] OR Norwegian[lang]) AND ("2000/01/01"[PDat]: "2010"[PDat])). The corresponding search terms were used for CINAHL®. Articles published in English, German, French, Spanish, Catalan, Danish and Norwegian were considered for inclusion. Two reviewers independently checked titles and abstracts for relevance and, in a second step, eligible full-text articles for inclusion. Reference lists of the included articles were checked manually. In addition, we followed PubMed-indexed related citations of two included articles which have been published recently and which focus on different acute care destinations [10,15].

We included prospective and retrospective, experimental and non-experimental studies if they 1) investigated residents from any type of LTC setting who were transferred to hospital emergency departments or hospital wards, 2) provided or assessed diagnostic and/or therapeutic data on the process of transfer, 3) developed, administered or derived a tool for assessing appropriateness of hospital admissions, including any list of aspects or any single question that could be used to distinguish between appropriate or inappropriate admissions. Studies using different terms (e.g. inappropriate, preventable, avoidable admissions) and operational definitions of appropriateness were considered for inclusion.

Two pairs of independent researchers extracted information on the study characteristics and the assessment tools using a piloted data extraction form. Publications cited in the reference list were retrieved if necessary. Results were discussed and, in the case of disagreement, a third author was consulted to reach consensus. In case of doubt, the authors of the primary study were contacted.

Data extraction covered information about the type of study, description of participants and settings, information on which assessment tool was used, how and by whom it was used, number and proportion of inappropriate admissions to acute care reported, period of time studied, and information on how the assessment tool was developed and which items were evaluated by the tool. Once data extraction was finished, the research team agreed on a list of aspects that were covered by the items found in the assessment tools.

We refrained from formal critical appraisal of the included studies, since we were interested in the concepts and tools used for assessing appropriateness of hospital admissions only, rather than the internal validity of the studies. Assessment of risk of bias would not have provided any substantial information with regard to the aim of this review.

Inter-rater reliability was not calculated because most information extracted was descriptive. All disagreements could be solved after checking for accuracy and discussion.

## Results

**Figure 1:**
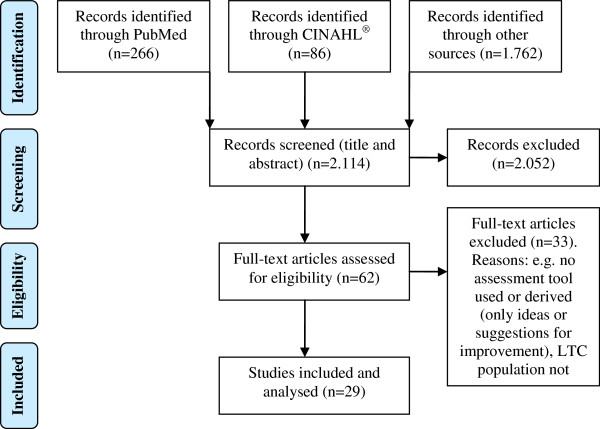
Identification of studies for inclusion in the systematic review.

Twenty-nine articles met the inclusion criteria [3,4,8-10,15,18-41]. Two articles reporting on the same study were considered as one source [21,38]. A list of studies excluded, along with the reason for exclusion, is available from the authors on request. Figure 1 displays the process of identification of studies for inclusion in the systematic review. (Additional file 1: Table S1) presents the characteristics of the included studies. The majority (n = 24) were retrospective. Five studies reported on an intervention or a strategy for reducing transfers to acute care (information not shown in the table) [21,23,26,27,30,38].

The majority of the studies (n = 24) investigated residents of LTC facilities only; five studies also included older persons living in the community [27,29,31,32,39]. Most studies (n = 25) considered the general population of LTC residents; four studies focused on specific groups: residents with long-term neurological conditions [32], residents with advanced cognitive impairment [37], and residents at the end-of-life [31,39]. Mean age of the study samples ranged from 81 [31,41] to 86 years [37], and the proportion of women varied from 62% [15] to 80% [23].

While types of LTC facilities seemed to be similar, the acute care destinations varied substantially: some studies focused either on ED visits or in-patient hospitalisation (n = 3), others included in-patient hospitalisation only, irrespective of a previous ED visit (n = 8), others included ED visits with consecutive in-patient hospitalisation (n = 2), ED visits with subsequent discharge to nursing homes (n = 1) or ED visits irrespective of subsequent in-patient hospitalisation (n = 6). Some studies investigated hospitalisation without any further specification (n = 9).

In eighteen studies the assessment tool used for determining appropriateness was applied to administrative databases. In eleven studies hospital or LTC facility records, or interviews with residents or nursing staff were used as data sources.

Results regarding the rate of inappropriate hospital admissions varied substantially. Some studies reported low proportions of inappropriate admissions. For example, Bermejo et al. [35] and Finn et al. [3] reported on 1.6% and 13.1% of inappropriate emergency department visits, respectively; Becker et al. [33] reported on 18% of preventable hospitalisation. Other studies documented high proportions of inappropriate admissions. In the study by Saliba et al. [18], 36% of all ED visits were judged as inappropriate; Walker et al. [19] and Ouslander et al. [30] reported on 55% and 77% of potentially avoidable hospitalisation, respectively.

Sixteen assessment tools determining appropriateness of hospital admissions among residents of LTC facilities were identified throughout the included studies. Information on their names, development, psychometric properties, aim/concept studied, way of use, items included and aspects covered are displayed in (Additional file 2: Table S2). Those tools without an own name are given the name of the first author of the corresponding study (see column “Tool [corresponding studies]”).

The terms used for indicating “inappropriate” hospitalisation varied throughout the different assessment tools: while most of them favoured the term “appropriate”/“inappropriate” (e.g., AEP), others used the terms “avoidable” or “preventable” (e.g., ACSC; additional tool by Finucane et al. [9]; AHC), and one study applied the term “potentially burdensome” (tool by Gonzalo et al. [37]).

Most tools aimed at measuring appropriateness of hospital transfer, i.e., from the LTC facility to either ED or hospital ward. Some of them focused on visits to ED (e.g., Modified AEP, tool by Jensen et al. [15]), while others focused on admissions to hospital (e.g., AEP), or on both ED visits and hospital stay (e.g. Quality Improvement Review tool (INTERACT-II)). A smaller number of tools aimed at determining those hospital transfers which could have been prevented by adequate ambulatory care (e.g., ACSC, AHC), focusing therefore on the period preceding the acute moment of transition.

All assessment tools were developed and based upon expert opinion to different extents: two tools were compiled using an expert consensus method, and six expert groups also took into account the results of a literature search or the interpretation of patient data. In all studies, tools were applied retrospectively, i.e., after hospital admission had already taken place.

Assessment tools were applied by the investigators themselves (n = 9), an external panel of experts (generally with experience in LTC) looking for consensus (n = 5), or professionals directly engaged in the care of residents transferred (n = 2).

As can be seen in Additional file 2: Table S2, some tools (e.g. AEP; ACSC) comprised a list of conditions or diseases (e.g. congestive heart failure, hypoglycaemia) while others consisted of a short definition or question (e.g. tool by Ong et al. [39], tool by Hammond et al. [32]).

The assessment tools differed widely regarding the aspects considered as criteria for judgement of appropriateness of acute care admissions. The six aspects are summarized in Table 1. Eight tools considered specific medical diagnoses as indicators for appropriate or inappropriate hospitalisation; seven tools considered the acuteness or severity of the symptoms at the moment of hospital transfer or admission; six tools took into account the resident’s characteristics prior to admission; three tools considered the residents’ or families’ wishes; one tool assessed whether a nursing care plan had been defined and adhered to; ten tools considered resource availability or requirement.

**Table 1:** Aspects covered by the assessment tools

**Aspect**	**Examples of items included in the tools**	**Number of tools covering the aspect**
Specific medical diagnoses	Suspected fracture, ACSC (asthma, congestive heart failure, angina, grand mal seizure disorder, hypoglycaemia, hypertension, etc.), death	8
Acuteness or severity of symptoms at time of transition	Sudden onset of unconsciousness, incapacitating pain, tachycardia, gastrointestinal bleeding symptoms, signs of being systemically unwell	7
Resident’s characteristics prior to admission to hospital	Resident’s baseline health status, level of functional ability, resident with advanced cognitive impairment, presence of a terminal illness	6
Resource availability/requirement	Requirement of intravenous antibiotics, laboratory, radiology, admission to hospital, physician and nurse availability and expertise	10
Residents’/families’ wishes	Advance care directive in place, request of hospital admission or emergency department visit by family	3
Information on the existence of a care plan	Actions taken by staff before the transfer (including presence of advanced care planning)	1

While most tools judged appropriateness based on one fulfilled item of the above mentioned aspects, five tools determined appropriateness by considering a balance of issues, for example by asking the professionals applying the criteria to give their judgement on appropriateness after considering all the aspects.

Some tools focused on one or two of the aspects (e.g. ACSC; tool by Gonzalo et al. [37]), while others were more comprehensive, i.e. covered a higher number of aspects. Six tools covered four aspects or more (e.g. tool by Abel et al. [31]; tool by Jensen et al. [15]; Quality Improvement Review tool; SIR).

Most tools (n = 10) were developed or adapted in the context of the actual studies, providing no information about their use in other studies or generalizability. Other tools had been used previously, but with an aim other than assessing appropriateness of admission to hospital (e.g. AEP). Finally, some tools had been developed or used only in one country or context (e.g. ACSC, Quality Improvement Review tool (INTERACT-II)). Moderate to good levels of inter-rater reliability were found for six tools (SIR; AEP; tool by Abel et al. [31]; tool by Hammond et al. [32]; tool by Codde et al. [34].

## Discussion

We reviewed 29 studies applying 16 assessment tools aimed at determining the appropriateness or preventability of ED or hospital admissions of LTC residents.

The rates of admissions considered as inappropriate differed substantially throughout the studies from 2% [9] to 77% [30]. The studies included in our review, most of them retrospective in nature and thus susceptible for bias, were distinctive in many aspects. They varied considerably in study designs and objectives. Outcomes were defined in different terms or even different concepts, e.g., inappropriate, avoidable, or preventable admissions. Besides, the acute care destinations varied, as well as the selection of the LTC population and LTC facility-level factors. Furthermore, studies took place in different regions and countries, implicating different reimbursement policies and financial incentives. The impact of these varying aspects on the rate of hospital admissions has been a matter of discussion for nearly 30 years. However, literature on this issue is scarce. In a previous review, case mix differences representing LTC population-level factors turned out to give only partial explanation for the variations in hospital admission [42]. This was confirmed by a study published by Wennberg et al., reporting that disparities in hospital admissions remained in similar geographic areas even after adjusting for case mix [43]. A recently published review of the literature confirmed that the propensity of being referred to acute care was rather associated with facility characteristics including nursing home ownership and bed-hold requirements than with patient characteristics [11].

Interestingly, to the best of our knowledge, the impact of assessment tools on the variability of inappropriate hospital admissions has not been studied so far.

In our review, we noticed considerable heterogeneity among the tools regarding the aims of use and the concepts studied (e.g. assessment of appropriateness of ED visits vs. in-patient hospitalisation; focus on preventable nature of the admissions vs. appropriateness of hospital transfer), format of use (tool applied by study authors vs. expert panel or nursing staff), data sources used (administrative databases vs. resident’ hospital or LTC facility record vs. interview with residents or nursing staff), and aspects evaluated.

Our research team isolated six most prominent aspects considered by the assessment tools: specific medical diagnoses, acuteness or severity of symptoms at transition time point, resident’s characteristics prior to admission to hospital, resource availability/requirement, residents’/families’ wishes, information on the existence of a care plan. Most tools covered less than four aspects, and only six of them included four or more aspects and were therefore considered as more comprehensive. The individual aspects “residents’ characteristics prior to admission to hospital” and “residents’/families’ wishes” were evaluated only by six and three tools, respectively. Some tools (e.g. ACSC, Modified ACSC) only evaluated aspects like “specific medical diagnoses” or “acuteness or severity of symptoms at transition time point”. Taking into consideration that residents in LTC facilities often differ in terms of comorbidity, cognitive and functional status, and stage of their diseases, it is surprising that residents’ clinical characteristics prior to acute care admission were not acknowledged throughout as a necessary dimension of the judgement process. The same applies to residents’ and relatives’ preferences which otherwise play an important role regarding the present advocacy towards person-centred care [44]. It may also be seen as a weakness of the existing tools that they did not consistently include facility-level characteristics as an indicator of the appropriateness of admissions. In respect to the frequently quickly changing conditions of residents, the presence of skilled nursing staff and the availability of technical equipment including diagnostic and therapeutic procedures may greatly influence the decision on the appropriateness of acute care admission. Finally, only 5 tools judged appropriateness based on a balance of aspects.

All tools identified in this systematic review were developed based on expert opinion, at least to a great extent. Information on generalizability in other regions or countries is scarce.

Our findings are supported by a non-systematic review [17,45]. Ouslander and Maslow did not focus on LTC residents only, but also included community-dwelling older persons. The review on preventable hospitalisations focusses on U.S. information sources and perspectives. The authors emphasize, as we do, the need for comprehensive measures to account for aspects such as medical comorbidities, clinical complexity or differences in resources in the care settings. They also criticize the lack of attention to how and where decisions about hospitalisation are made.

Our systematic review focussed on the assessment of appropriateness among LTC residents. The assessment of appropriateness of hospital admission among community-dwelling older persons may require the consideration of similar aspects, but adapted to the different setting. To the best of our knowledge, no systematic review covering international studies on this issue is available so far.

It may be seen as a limitation that we did not systematically assess the risk of bias of the original studies included in our systematic review. However, we were interested in the concepts and tools used for assessing appropriateness of hospital admissions, rather than in the internal validity of the studies. Nevertheless, even without formal validity assessment, it is obvious that the included studies suffer from methodological shortcomings, since many used secondary or retrospective routine data analysis and are therefore more prone to bias.

Our review, which is the first to overview the tools internationally used to assess the appropriateness of hospital admissions among LTC residents, may contribute to the clarification of the concept “appropriateness of admission of LTC residents to acute care”. It also may present a first step towards the development of an evidence-based, comprehensive and generalizable tool. Such a tool may have a two-fold function: first as a quality indicator to assess the appropriateness of the decisions made when admitting individual residents to acute care, considering that the resources available were not modifiable at that time, and secondly to identify areas of improvement such as the need for training in palliative care or the need for more resources. The tool may attempt to assess appropriateness minimizing the effects of the different rater perspectives (i.e. nursing staff of the LTC facility, ED professionals, and researchers). It may also be used to assess the effectiveness of new interventions aimed at improving appropriateness of transition of LTC residents to acute care.

In the meanwhile, studies aiming at assessing appropriateness of admitting LTC residents to hospital are encouraged to use an assessment tool according to predefined aims and taking the different aspects into consideration. Studies should mention why a certain tool was chosen and the limitations of not using a more comprehensive tool should be clearly mentioned.

## Conclusions

Our systematic review analysed 29 studies assessing the prevalence of the appropriateness of acute care admissions, which varied widely throughout the studies. We found 16 different assessment tools used in the studies. Only six tools covered more than four aspects as criteria to determine the appropriateness of acute care admissions. Most assessment tools did not take into account residents' individual aspects, such as characteristics of residents prior to admission and wishes of residents or families. Tools were based mostly on expert opinion, and information on their generalizability is not provided. Further research is warranted to develop an evidence-based and comprehensive tool supported by quality assuring strategies to improve decisions on the appropriateness of ED and hospital admissions among residents of LTC facilities.

## Competing interests

The authors declare that they have no competing interests.

## Authors’ contributions

Review protocol: ARG, EM & GM. Literature search: ARG, EM, LU & GM. Data extraction: ARG, EM, LU & GM. Data interpretation: ARG, GM, EM, LU. Drafting of the manuscript: ARG. Critical revision of the manuscript with regard to important intellectual content: GM, EM & LU. Study supervision: EM & GM. All authors read and approved the final manuscript.

## Pre-publication history

The pre-publication history for this paper can be accessed here:

http://www.biomedcentral.com/1471-2318/14/80/prepub

## Supplementary Material

Additional file 1: Table S1 Studies dealing with assessment tools for determining appropriateness of hospital admissions among residents of LTC facilities. Click here for file

Additional file 2: Table S2 Characteristics of the assessment tools to determine appropriateness of hospital admissions among residents of LTC facilities [46]- [35]. Click here for file
