# Supplementary material for: Assessment tools for determining appropriateness of admission to acute care of persons transferred from long-term care facilities: a systematic review
Source: BMC Geriatr. 2014 Jun 22;14:80. doi: 10.1186/1471-2318-14-80 (PMC4094601; doi:10.1186/1471-2318-14-80)
Supplement: Additional file 1: Table S1 — Studies dealing with assessment tools for determining appropriateness of hospital admissions among residents of LTC facilities. [file 1471-2318-14-80-S1.docx]

**Additional file 1: Table S1:** Studies dealing with assessment tools for determining appropriateness of hospital admissions among residents of LTC facilities.

| **Authors and publication year** | **Country** | **Studied period** | **Method** | **Sample^a^** | **Type of LTC facilities and number** | **Acute care destination and number of facilities** | **Outcome concept and number or % of inappropriate admissions** | **Assessment tool used and data source (administrative databases, resident hospital / LTC facility records, interview)** |
| --- | --- | --- | --- | --- | --- | --- | --- | --- |
| **Saliba et al., 2000** [18] | USA | 1994-1995 | Retrospective, secondary data analysis | Residents admitted to hospital (n=458) | NH (n=8) | EDV or IH (n=10) | Inappropriate EDV: 36% of the admissions; Inappropriate IH: 40% | SIR (LTC facility and hospital records) |
| **Finucane et al., 2000** [9] | Australia | 1998 | Prospective, observational study | Residents admitted to hospital (n=153), admissions (n=184) | NH, rest homes, hostels (NNM) | EDV and consecutive IH (n=1) | Inappropriate hospitalisation: all participants 2/184 (1%), NH residents 1/65 (2%). Potentially avoidable hospitalisation : all 19/184 (10%), NH 17/65 (26%) | AEP + additional question on avoidability (LTC facility and hospital records, interview) |
| **Murtaugh, 2002** [22] | USA | 1992-1994 | Retrospective, secondary data analysis | Older persons, including home care patients (n=3.057) | LTC settings (NNM) | IH (NNM) | Avoidable hospitalisation: 2% of the IH (not specific data for NH participants reported) | AHC (administrative databases) |
| **Kane et al., 2003** [23] | USA | 1998-2000 | Retrospective, routine data analysis | Residents: intervention group (n=1.936), control groups (n=2.868) | Control and intervention NH (n=44 pairs) | IH (excluding IH after EDV) (NNM) | Preventable hospitalisation per 100 residents (rate per month): intervention 0.3; control I 0.8 (p<0.001); control II 0.9 (<0.001) | ACSC (administrative databases) |
| **Carter, 2003** [24] | USA | 1991-1993 | Retrospective, secondary data analysis | Residents admitted to hospital (n=72.319 person-quarter observations) | NH (n=527) | Hospitalisation^b^ (NNM) | Preventable hospitalisation due to ACSC: n= 8.070 (11%) | Modified ACSC (administrative databases) |
| **Intrator et al., 2004** [25] | USA | 1997 | Prospective, observational, cross-sectional | Residents (n=54.631) | NH (663) | Hospitalisation^b^ (NNM) | Potentially preventable or avoidable hospitalisation: n=3.137 (37%) out of 8.450 hospitalised at least once | ACSC (administrative databases) |
| **Kane et al., 2004** [26] | USA | 1998-2000 | Retrospective, routine data analysis | Residents: intervention group (n=1.936), control groups (n=2.868) | Control and intervention NH (n=44 pairs) | IH (excluding IH after EDV) (NNM) | Preventable hospitalisation per 100 residents (rate per month): intervention 0.4; control I 0.9 (p<0.001); control II 1.1 (<0.001) | ACSC (administrative databases) |
| **Kane et al., 2005** [27] | USA | 1997-2001 | Retrospective, routine data analysis | Residents: intervention group (n=1.985), control groups (n=3.970) | Control NH (n=181-289), intervention NH (n=110-118) (3 studied periods) | EDV or IH (NNM) | Preventable hospitalisation per 100 residents (rate per month): intervention 0.4; control I 0.7; control II 0.6.  Preventable EDV per 100 residents (rate per month): intervention 1.7; control I 2.6; control II 2.3 | Modified ACSC (administrative databases) |
| **Carter and Porell, 2005** [28] | USA | 1991-1993 | Retrospective, secondary data analysis | Residents with ADRD (n=19.802), residents without ADRD (n=19.958) | NH (n=525) | IH (NNM) | Avoidable hospitalisation or hospitalisation due to ACSC: 41% of the IH among residents with ADRD; 43% of the IH among those without ADRD | ACSC (administrative databases) |
| **Finn et al., 2006** [3] | Australia | 2002 | Retrospective, routine data analysis | Admissions to hospital from residential care institutions (n=541) | NH, hostels (NNM) | EDV (n=1) | Inappropriate EDV: n=71 (13%) | Modified AEP (resident hospital records) |
| **Carter et al., 2006** [29] | US | 2000-2002 | Retrospective, secondary data analysis | Admissions to hospital from NH (n=1.279) | NH (NNM) | EDV and consecutive IH (NNM) | Potentially avoidable EDV and consecutive ICH with ACSC^c^ | Modified ACSC (administrative databases) |
| **Grabowski et al., 2007** [8] | USA | 1998-2004 | Retrospective, routine data analysis | Residents: 1999 (n=167.452), 2000 (n=165.228), 2001 (n=162.946), 2002 (n=161.967), 2003 (n=161.726) | NH (n=690) | IH (n=253) | IH with ACSC in 1999: 34%; 2000: 33%; 2001: 32%; 2002: 32%; 2003: 30%; 2004: 29% | ACSC (administrative databases) |
| **Jensen et al., 2009** [15] | Canada | 2000 | Retrospective, routine data analysis | Residents admitted to hospital (n=606) | LTC facilities (n=19) | EDV (n=3) | Inappropriate EDV: n=2 (4%) | In-house developed (resident hospital records) |
| **Walker et al., 2009** [19] | Canada | 1997-2002 | Retrospective, routine data analysis | Residents (n=76.629); Residents admitted to hospital (n=8.885) | High intensity LTC facilities (n=150) | Hospitalisation^b^ (NNM) | Potentially avoidable hospitalisation according to the original US ACSC list: 47% (n=4.177 out of 8.885); according to the revised Canadian list: 55% of hospitalisation (n=4.874 out of 8.885) | Modified ACSC (administrative databases) |
| **Ouslander et al., 2009** [30] | USA | 2005-2007 | a) Retrospective, routine data analysis; b) prospective, interventional pilot single arm study. Comparison of both data sets | a) Residents admitted to hospital (n=30); b) Residents admitted to hospital (n=65) | NH (n=3) | Hospitalisation^b^ (NNM) | Potentially avoidable hospitalisation: a) n=23 (77%); b) n=32 (49%) | Modified SIR (resident hospital and LTC facility records) |
| **Abel et al., 2009** [31] | England | 2006-2007 | Retrospective, routine data analysis | Residents admitted to hospital from NH (n=77) and RH (n=59) (who died in this episode of care) | NH, RH (NNM) | IH (irrespective of EDV) (n=1) | Appropriateness of staying at the LTC facility yes/maybe (inappropriately transferred): NH n=53 (69%); RH n=27 (45%) | In-house developed (resident hospital records) |
| **Hammond et al., 2009** [32] | UK | 2006-2007 | Prospective | Residents with LTNC admitted to hospital (n=25) | NH (NNM) | IH (n=2) | Inappropriateness of admission: 12% (3 out of 25) | In-house developed (resident hospital records and structured interviews with residents) |
| **Gruneir et al., 2010** [4] | Canada | 2005 | Retrospective, secondary data analysis | Residents (n=64.589) | NH (NNM) | EDV (NNM) | Potentially avoidable EDV: 25% of all EDV | ACSC (administrative databases) |
| **Ouslander et al., 2010** [10] | USA | 2005-2006 | Retrospective, routine data analysis | Residents admitted to hospital (n=200) | NH (n=20) | Hospitalisation^b^ (NNM) | Probably or definitely avoidable hospitalisation: n=134 (67%) | Modified SIR (resident LTC facility records) |
| **Becker et al., 2010** [33] | USA | 2003-2006 | Retrospective, routine data analysis | Residents (n=72.251); residents admitted to hospital (n= 8.382) | NH (n=647) | Hospitalisation^b^ (NNM) | Preventable hospitalisation: 18% of all hospitalisation (n=10.091 out of 8.382) | ACSC (administrative databases) |
| **Caffrey, 2010** [20] | USA | 2004 | Retrospective, secondary data analysis | Residents (n=14.017) | NH (n=1.500) | EDV (NNM) | Potentially preventable EDV: 40% among residents with an EDV | Adapted from INTERACT II and other sources (administrative databases) |
| **Codde et al., 2010** [34] | Australia | 2007 | Retrospective, routine data analysis | Residents admitted to hospital and discharged to NH (n=235) | NH (NNM) | EDV and discharge to NH (n=1) | Potentially avoidable EDV: 161 (69%) of patients discharged to NH; 31% of the total transfers, including patients with IH | In-house developed (resident hospital records) |
| **Bermejo et al., 2010** [35] | Spain | 2008 | Retrospective, routine data analysis | Residents admitted to hospital (n=45); admissions to hospital (n=62) | NH (n=1) | EDV (n=1) | Inappropriate or not suitable EDV: 2% of all EDV | In-house developed (resident hospital and LTC facility records) |
| **Kada et al., 2011** [36] | Austria | 2008 | Retrospective, routine data analysis + qualitative interviews | Residents admitted to hospital (n=4.149);  residents with EDV (n=423) | NH, RH (n=15) | EDV (n=1) | Inappropriate EDV: 22% of all EDV | Modified AEP (administrative databases) |
| **Gonzalo et al., 2011** [37] | USA | 2000-2007 | Retrospective, routine data analysis | Residents with ACI admitted to hospital (n=474.829) | NH (NNM) | Hospitalisation^b^ (NNM) | Potentially burdensome transition to acute care: 6% of the residents with ACI | In-house developed (administrative databases) |
| **a) Ouslander et al., 2011** [38]**;**  **b) Lamb et al., 2011** [21] | USA | 2008-2009 | a) Prospective, single arm intervention; comparison with retrospective data;  b) prospective single arm intervention + one-hour conference calls | a) Residents per NH (average size n=166); b) Residents per NH (average size n=174) | a) NH (n=25); b) NH (n=26) | EDV, IH (NNM) | Avoidable or possibly avoidable hospitalisation: 24% of hospitalisation (b) | Quality Improvement Review tool (INTERACT-II) (resident LTC facility records, and written questions to nursing staff) |
| **Ong et al., 2011** [39] | England | 2005-2006 | a) Retrospective, routine data analysis + b) prospective qualitative analysis | Residents admitted to hospital from RH (n=223) and NH (n=117) | a) NH, RH (NNM); b) NH, RH (n=8) | IH (n=1) | Potentially avoidable or inappropriate acute hospitalisation (likely to have been managed in care homes): 41% of hospitalisation | In-house developed (administrative databases) |
| **Young et al., 2011** [40] | USA | 2006-2007 | Retrospective, routine data analysis and secondary data analysis | Residents (n=26.746) | NH (n=147) | Hospitalisation^b^  (NNM) | Potentially preventable hospitalisation due to ACSC rate: 654 per 100.000 resident-days | ACSC (administrative databases) |
| **Becker et al., 2012** [41] | USA | 2002-2008 | Retrospective, secondary data analysis | Residents (n=16.208); residents older than 65 years (n=7.991) | Assisted living facilities | Hospitalisation^b^  (NNM) | Hospitalisation due to ACSC: 22% (among residents older than 65 years) | ACSC (administrative databases) |
| Note: ACI: Advanced Cognitive Impairment; ACSC: Ambulatory Care Sensitive Conditions; ADRD: Alzheimer’s Disease and Related Dementias; AEP: Appropriateness Evaluation Protocol; AHC: Avoidable Hospital Conditions; EDV: Emergency Department Visit; IH: In-patient Hospitalisation; ISD: Intensive Service Days; LTC: Long Term Care; LTNC: Long Term Neurological Conditions; NH: Nursing Home; NNM: Number Not Mentioned; RH: Residential Home; SIR: Structured Implicit Review.  ^a^Only data from LTC facilities are displayed, if available.  ^b^Not specified if EDV or IH.  ^c^Number of inappropriate admissions not provided. | | | | | | | | |
