# Supplementary material for: Assessment tools for determining appropriateness of admission to acute care of persons transferred from long-term care facilities: a systematic review
Source: BMC Geriatr. 2014 Jun 22;14:80. doi: 10.1186/1471-2318-14-80 (PMC4094601; doi:10.1186/1471-2318-14-80)
Supplement: Additional file 2: Table S2 — Characteristics of the assessment tools to determine appropriateness of hospital admissions among residents of LTC facilities [46]- [35]. [file 1471-2318-14-80-S2.docx]

**Additional file 2: Table S2:** Characteristics of the assessment tools to determine appropriateness of hospital admissions among residents of LTC facilities.

| **Tool [corresponding studies]** | **Term(s), concept(s) and aim(s) of use** | **Development** | **Psychometric properties** | **Format of use in the included studies** | **Summary of the items evaluated (aspects covered^a^)** |
| --- | --- | --- | --- | --- | --- |
| **SIR** [10,18,30] | To measure agreement between reviewers on the appropriateness of decisions to transfer NH residents to EDs or hospital [18], frequency and reasons for potentially avoidable hospitalisations [10], and efficacy of strategies to reduce potentially avoidable hospitalisation [30] | Developed by Saliba et al., 2000 [18]. Based on medical literature and semi-structured interviews with professionals and experts (e.g. nursing facility administrators, geriatric nurse practitioners, emergency room physicians, family medicine physicians, and geriatricians). Modified by Ouslander et al., 2010 [10] | Reviewer agreement: 84% agreement for EDV (kappa 0.68) and 89% and for hospitalisation [18] | Implicit criteria: list of questions for trained reviewers (experienced in LTC). Afterwards, they had to answer the question "was the hospitalisation avoidable?" with: "definitively not", "probably not", "probably yes" or "definitively yes" | Balance of issues between: residents’ baseline health status (C), advance directives (W), potential benefits of acute transfer (R), and the care provided in the NH when the residents’ status changed (R). |
| **AEP** [9] | To measure appropriateness of admission to hospital | Mostly expert based. Original version by Gertman and Restuccia, 1981 (USA), used to assess potentially unnecessary hospital days of care (not specific between NH and acute care) [46]. Refined by Baggoley et al., 1994 [47] | Original AEP (German and Restuccia, 1981) Overall agreement: 92% to 94% (p<0.0001); specific agreement rates for the reviewer pairs: 73% to 79% | List of items applied to residents' data by the authors. Hospitalisations deemed appropriate if any criteria fulfilled. | Items indicating appropriateness: 1) Severity of illness (e.g. sudden onset of unconsciousness, abnormally high or low pulse rate, persistent fever, incapacitating pain, electrocardiogram abnormality) (A); 2) Intensity of service (e.g. parenteral medications and/or fluid replacement, vital sign monitoring) (R) |
| **Modified AEP** [3,36] | To measure appropriateness of EDV | Defined by an expert multidisciplinary clinical review panel. Modified by Finn et al., 2006 [3] | No data provided | List of criteria applied by a research study nurse to the medical records of participants. Records of patients not meeting the criteria reviewed by a clinical panel (consisting of different professionals from both acute care and LTC) to determine whether the episodes could have been managed within the nursing homes. | Items indicating appropriateness, e.g. procedure unable to be performed in a nursing home (R), history of trauma with suspected fracture (D), difficult indwelling catheter insertion (R), PEG tube insertion (R), suspicion of cerebral event with decreasing consciousness (A), requirement for intravenous antibiotics (R), admission to hospital (R) |
| **Additional tool^b^** [9] | To identify potentially avoidable hospitalisation | Developed in the context of the study as additional tool to AEP [9]. Methods not specified. | No data provided | Additional question to AEP. Applied by authors. Case conference involving senior clinicians | Availability of specialised care (e.g. parenteral fluid, parenteral drugs, high level of medical and nursing supervision) within the residential care setting (R) |
| **AHC** [22] | To identify potentially avoidable hospital stays, defined as hospital admissions for conditions suggesting inadequate ambulatory care | Developed in the context of the study [22]. Literature review and expert opinion considered. Based on research from Weissman et al., 1992 [48] | No data provided | List of items applied to residents' data by the authors | Items indicating avoidability, e.g. heart failure, urinary tract infection (D) |
| **ACSC** [4,11,23-29,33,40,41] | To identify preventable EDV or potentially avoidable hospitalisation of NH residents | Developed in the context of Billings et al., 1993 [49]. Modified Delphi method including a medical advisory panel of six internists and paediatricians, including national and local experts. Originally developed for community-dwelling older adults. Several modifications exist [50,24] | No explicit data found | List of items applied by the authors to residents' data | Items indicating avoidability, e.g. asthma, congestive heart failure, angina, grand mal seizure disorder, hypoglycaemia, hypertension. Modifications, e.g. Carter (2003) excluded pneumonia and congestive heart failure [24]; Kane (2005) added accidents and poisonings to the preventable emergency services [27] (D) |
| **Modified ACSC** [19] | To identify potentially avoidable hospitalisation in LTC facilities and to identify opportunities for improvement in preventive care, provider continuity and chronic disease management | Developed in the context of the study [19]. Expert panel assessed applicability of the pre-existing ACSC to an older institutionalised population in Canada and developed consensus-based revisions appropriate for the setting | No data provided | List of items applied to residents' data by the authors | Two items added to ACSC: septicaemia and falls/fractures; four conditions deleted: immunization-preventable conditions, nutritional deficiency, severe ear, nose and throat infections, tuberculosis (D) |
| **Tool^b^ by Jensen et al., 2009**  [15] | To assess appropriateness of EDV of LTC residents | Developed in the context of the study [15]. Defined by a physician team experienced in LTC (a health researcher and family physicians) | No data provided | Physician team (experienced in LTC) independently reviewed resident cases and made clinical judgment on appropriateness of referral. Consensus meeting. | Appropriateness defined as a balance of issues: timeliness, availability of diagnostic and treatment resources (e.g., intravenous, pharmaceuticals) (R), timely test results (R), physician availability and expertise (R), nursing availability and expertise (R), advanced directives (W), respect for patient and family wishes (W), availability of history and medical information, premorbid health status (C) |
| **Tool^b^ by Abel et al., 2009**  [31] | To measure the appropriateness of staying at home (or LTC facility).  Specific for the end-of-life phase. | Developed by authors, based on a previously developed national strategy: “End of Life Strategy” (Department of Health 2008), which considers the best existing evidence [51] | Level of agreement between consultants: kappa range 0.59, 0.70 | One author (consultant for palliative medicine) reviewed the cases notes and applied the tool. Another author independently reviewed a random sample (10%). Appropriateness coded as "no" if it was clear that the resident needed hospital admission, "yes" if it was clear that they could have stayed at home and "maybe" if there was a degree of uncertainty. | Three aspects, balance of issues: 1) Assumption that the patient could have been looked after at home, if the End of Life Strategy (includes recognising patients as being in the last year of life, advance planning concerning place of death and priorities for care, care available at short notice 24 hours per day, nursing care at home available for final stages of life) was implemented and services available (R); 2) The patient should have a terminal illness as described in the Gold Standards Framework Prognostic Indication Guidance (C); 3) The cause of admission should not require immediate inpatient medical attention (A) |
| **Tool^b^ by Hammond et al., 2009** [32] | To measure the appropriateness of admissions and IH for patients with LTNC. To identify management alternatives for inappropriate admissions | Developed in the context of the study [32]. Methods not specified, probably based on expert opinion | Inter-rater reliability referring to agreement in judging the appropriateness of admission: kappa range 0.42- 0.44,  Intra-rater reliability referring to the agreement between individuals’ baseline decision and overall panel decision: 79%- 90% of cases | Panel of experts (a neurological rehabilitation physician, an acute care physician and a general practitioner) reviewed the cases notes and used the working definition to decide on appropriateness. Consensus meeting. | Working definition: "admissions deemed appropriate when the level of care required can only be provided at the hospital e.g. access to specialist equipment required, treatment administration such as intravenous antibiotics, or urgent specialist input". Data on medical history (C), admitting problem (A), circumstances surrounding the admission (A/R), level of functional ability (C), dependence and cognitive status (C) used. Balance of issues. |
| **Tool^b^ by Caffrey, 2010** [20] | To measure potentially preventable EDV by NH residents | Authors took medical conditions included at the INTERACT II tool and added conditions from other studies | No data provided | List of items applied by the authors to the data | Items indicating preventability, e.g. general fever symptoms (A), general chest pain symptoms (A), heart disease symptoms (A), symptoms of mental status changes (A), gastrointestinal bleeding symptoms (A), urinary tract infection symptoms (A), metabolic disturbance diseases (D), pneumonia (D), diseases of the skin (D) |
| **Tool^b^ by Codde et al., 2010** [34] | To measure potentially avoidable EDV by applying indicators and exclusion criteria | Developed in the context of the study [34]. Combination of expert opinion and prior work from Finn et al., 2006 (Modified AEP) [3] | Inter-rater reliability: 0.41, (95% CI 0.28-0.56) | List of items applied by the authors to the data | Items indicating avoidability, e.g. assessment and simple wound dressing or closure required (R), uncomplicated UTI (D), replacement of gastrostomy tube (R), advance care directive in place (W); Exclusion criteria for potentially avoidable conditions, e.g. triaged as category one on arrival in ED (A), trauma with suspected long bone fracture (D), laboratory or radiology necessary (R), signs of being systemically unwell (A), significant neurological changes (A), intravenous medication required (R), family requested ED (W) |
| **Quality Improvement Review tool (INTERACT-II)** [21,38] | To measure avoidability of EDV or IH of NH residents according to the NH staff; to assist NH staff in understanding the reasons for the transfer, identify opportunities to improve identification and management of changes in resident status, and reduce acute care transfers | Part of INTERACT II tool, based on analyses of data on hospitalisations rated by experts as potentially avoidable and on expert recommendations on the feasibility and importance of a variety of interventions | No data provided | Questionnaire to be filled in by NH staff. Once they have evaluated all the items they are required to answer to the question: "In retrospect, does your team think this transfer might have been prevented?" with "no" or "yes" and to provide opportunities for improvement. | Balance of issues between: resident information (C), hospital transfer information, including symptoms or change in condition that precipitated the transfer (A), actions taken by staff before the transfer (including presence of advanced care planning) (R, P, W); analysis of factors that may have influenced the transfer decision. |
| **Tool^b^ by Bermejo et al., 2011** [35] | To measure the appropriateness or suitability of EDV | Developed by the authors using data from prior studies | No data provided | The authors reviewed the cases notes and applied the tool. | Appropriate EDV if one criteria fulfilled: 1) Patient admitted to a hospital ward or stayed in observation for more than 24 hours (R); 2) Specialist visit or diagnostic test required, not available in the LTC facility (R); 3) Requirement of a treatment not available in the LTC facility (R) |
| **Tool^b^ by Gonzalo et al., 2011** [37] | To measure potentially burdensome transitions among NH residents with advanced cognitive and functional impairment | Developed in the context of the study on the basis of a previously conducted narrative analysis with families of patients affected and expert opinion [52] | No data provided | The authors reviewed the cases notes and applied the tool. | Condition defining burdensome transition: any transfer to acute care hospital of a resident with advanced cognitive impairment (C) in the last 3 days of life (D) |
| **Tool^b^ by Ong et al., 2011** [39] | To measure avoidable or inappropriate acute hospitalisation of NH and RH residents | Method of development not specified | No data provided | The authors reviewed the cases notes and applied the tool. | Condition defining avoidability: patients dying within 3 days after hospital admission considered inappropriately transferred; patients dying (D) after 7 days considered appropriately transferred |
| Note: ACSC: Ambulatory Care Sensitive Conditions; AEP: Appropriateness Evaluation Protocol; AHC: Avoidable Hospital Conditions; CI: Confidence Interval; EDV: Emergency Department Visits; NH: Nursing Home; LTC: Long Term Care; LTNC: Long Term Neurological Conditions; PEG: Percutaneous Endoscopic Gastrostomy; RH: Residential Home; SIR: Structured Implicit Review; UTI: Urinary Tract Infection.  ^a^Aspects covered: A: acuteness/severity of the symptoms; C: resident's characteristics prior to admission to hospital; D: specific medical diagnoses; P: existence of a care plan; R: resource availability; W: residents’ or families’ wishes.  ^b^Tool without a specific name. | | | | | |
